# The impact and public health response of chiropractors to the COVID-19 pandemic: a survey across four continents

**DOI:** 10.1186/s12998-022-00432-6

**Published:** 2022-05-09

**Authors:** Craig Moore, Arnold Y. L. Wong, Katie de Luca, Diana De Carvalho, Melker S. Johansson, Katherine A. Pohlman, Amy Miller, Martha Funabashi, Paul Dougherty, Simon French, Jon Adams, Greg Kawchuk

**Affiliations:** 1 Department of Chiropractic, Faculty of Medicine, Health and Human Sciences, Macquarie University, Sydney, Australia; 2 Department of Rehabilitation Sciences, The Hong Kong Polytechnic University, Hung Hom, Hong Kong; 3 Memorial University of Newfoundland, St. John’s, Canada; 4 Department of Sports Science and Clinical Biomechanics, University of Southern Denmark, Odense, Denmark; 5 Department of Public Health, University of Southern Denmark, Odense, Denmark; 6 Research Center, Parker University, Dallas, USA; 7 AECC University College, Bournemouth, UK; 8 Division of Research and Innovation, Canadian Memorial Chiropractic College, Toronto, Canada; 9 Department of Chiropractic, Université du Québec à Trois-Rivières, Trois-Rivières, Canada; 10 VA Finger Lakes Healthcare System, Canandaigua, USA; 11 School of Public Health, Faculty of Health, University of Technology, Sydney, Australia; 12 Faculty of Rehabilitation Medicine, University of Alberta, Edmonton, Canada

**Keywords:** COVID-19, Infection control, Chiropractors, Behaviours, Telehealth

## Abstract

**Background:**

The unprecedented impact of COVID-19 on healthcare professionals has implications for healthcare delivery, including the public health guidance provided to patients. This study aims to assess the response and impact of COVID-19 on chiropractors internationally, and examines the public health response of chiropractors to the COVID-19 pandemic practising under a musculoskeletal spine-care versus subluxation-based care paradigm.

**Methods:**

A survey was distributed to chiropractors in Australia, Canada, Denmark, Hong Kong, United Kingdom and United States (Oct. 2nd–Dec. 22nd, 2020) via professional bodies/publications, and social media. Questions were categorised into three domains: socio-demographic, public health response and business/financial impact. Multivariable logistic regression explored survey items associated with chiropractors practising under different self-reported paradigms.

**Results:**

A total of 2061 chiropractors representing four global regions completed the survey. Our recruitment method did not allow the calculation of an accurate response rate. The vast majority initiated COVID-19 infection control changes within their practice setting, including increased disinfecting of treatment equipment (95%), frequent contact areas (94%) and increased hand hygiene (94%). While findings varied by region, most chiropractors (85%) indicated that they had implemented regulator advice on the use of personal protective equipment (PPE). Suspension of face-to-face patient care during the peak of the pandemic was reported by 49% of the participants with 26% implementing telehealth since the pandemic began. Chiropractors practising under a musculoskeletal spine-care paradigm were more likely to implement some/all regulator advice on patient PPE use (odds ratio [OR] = 3.25; 95% confidence interval [CI]: 1.57, 6.74) and practitioner PPE use (OR = 2.59; 95% CI 1.32, 5.08); trust COVID-19 public health information provided by government/World Health Organisation/chiropractic bodies (OR = 2.47; 95% CI 1.49, 4.10), and initiate patient telehealth in response to COVID-19 (OR = 1.46; 95% CI 1.02, 2.08) compared to those practising under a subluxation-based paradigm.

**Conclusions:**

Chiropractors who responded to our survey made substantial infectious control changes in response to COVID-19. However, there was regional variation in the implementation of the advised practitioner and patient use of PPE and limited overall use of telehealth consultations by chiropractors during COVID-19. Musculoskeletal spine-care chiropractors were more adaptive to certain COVID-19 public health changes within their practice setting than subluxation-based chiropractors.

**Supplementary Information:**

The online version contains supplementary material available at 10.1186/s12998-022-00432-6.

## Background

The unprecedented impact of COVID-19 on healthcare practitioners has been an important focus for public health [1, 2]. This has included implementing a range of infection control measures within clinal practice settings, such as the use of personal protective equipment (PPE) [3] and delivering public health information and advice to patients to help reduce the spread of COVID-19 [4]. While managing these responsibilities, practitioners have also faced substantial personal burden in managing the negative impacts of COVID-19 on their business and finances [5]. Recent research has provided important insights into the public health response and impact of the pandemic on various healthcare professions [5, 6]. Gathering this information can help to better understand the health services implications, public health literacy and personal burdens of COVID-19 on practitioners [7].

Chiropractors are regulated, primary healthcare professionals who make up a large international workforce [8] and predominantly deliver evidence-based care to individuals with musculoskeletal disorders [9]. In contrast, some chiropractors focus their care on the correction of spinal lesions, identified as ‘chiropractic subluxations’, with the aim of influencing nervous system communication to improve health and prevent disease [10]. Infection control standards for chiropractors vary according to country and specific jurisdictional mandates should be the best predictor of professional behaviour. However, limited information suggests chiropractors practising under a subluxation-based paradigm may hold different public health views to that of government health authorities and/or scientific consensus regarding COVID-19, including a belief that chiropractic adjustments (spinal manipulation) can boost the immune response to COVID-19 infection, improving protection against the disease [11–14].

To date, very little is known about the impact and response of chiropractors to the COVID-19 pandemic. In response to this gap, we surveyed chiropractors in Australia, Canada, Denmark, Hong Kong, the United Kingdom (UK), and the United States (US) to:

1. Describe the public health response of practicing chiropractors to COVID-19 within their practice setting;
2. Describe the impacts of the COVID-19 pandemic on the business and finances of practising chiropractors; and
3. To examine if the public health response to the COVID-19 pandemic of chiropractors self-reporting as practising under a musculoskeletal spine-care paradigm differs from those practicing under a chiropractic subluxation-based care paradigm.

## Methods

Ethical review was approved by the Macquarie University, Faculty of Science and Engineering Human Research Ethics Committee, approval number: 52020679320510. All data were collected, password protected and stored at Macquarie University.

### Procedures

An online questionnaire developed by a multidisciplinary research team (including chiropractors, epidemiologists, public health researchers and physiotherapists) was distributed to licensed and practising chiropractors through email communication channels of chiropractic organisations and outlets in Australia (Australian Chiropractors Association, Chiropractic Australia), Canada (Canadian Chiropractic Association), Hong Kong (Chiropractic Doctors Association of Hong Kong), Denmark (Danish Chiropractors’ Association), UK (British Chiropractors’ Association, General Chiropractic Council) and the US (Dynamic Chiropractic magazine). The questionnaire was distributed (October 2nd, 2020 and December 22nd, 2020) via the LimeSurvey electronic data capture platform. LimeSurvey was used via the university’s official subscription and hosted on secure local infrastructure to ensure the highest standards of privacy, confidentiality and data sovereignty are maintained. Our survey set a browser cookie to ensure participants did not complete the survey more than once. Two reminders to complete the survey were sent during the recruitment period. A link to the online questionnaire was further disseminated widely via Twitter, chiropractic-focused social media groups (Facebook) and email.

### Survey instrument

Key themes and survey questions were developed from other surveys evaluating the response and impact of the COVID-19 pandemic on primary care providers [5, 6]. The background page included the ethics board and researcher contact information, complaints procedures as well as information about the purpose and content of the survey, potential risks and benefits of participation, expected duration to complete the questionnaire, voluntary participation and confidentiality. All practitioners provided their informed consent electronically after reading the study background information and prior to participating in the study. Potential participants were informed that survey participation requires them to confirm their consent and understanding of the background information. A checkbox was used to allow them access to the survey questionnaire by confirming they are a licensed practising chiropractor and understand what they have read and consent to proceed as a participant (or to otherwise exit from starting the survey).

The questionnaire comprised three main sections with responses as multiple-choice items or ratings on a 4- or 5-point Likert scale (Additional file 1: Appendix 1). The first section collected practitioners’ sociodemographic information, including age, country, years in practice, institution/level of education and prevailing practice paradigm, henceforth reported as musculoskeletal spine-care or subluxation-based care (or neither). Respondents could only select one of the paradigm options which the respondent thought “more closely” described the care they provide and were not given the option to choose both paradigms. The second section collected information on the public health response of chiropractors to the COVID-19 pandemic within their practice setting including hand hygiene, disinfection of equipment, patient social distancing, use of PPE (chiropractors and patients), knowledge of government advice on the use of PPE, use of patient telehealth, providing COVID-19 public health information to patients and trusted resources utilised for COVID-19 public health information. The third section collected information on the impacts of COVID-19 on the business and finances of chiropractors, including changes in the level of face-to-face patient care, changes in personal income, need for financial assistance and impacts of COVID-19 on the employment of other practice staff and future concerns. Participants were required to fully complete the initial sociodemographic section (non-completion of this section prevented participants from progressing to the subsequent sections of the questionnaire to participate). For the remainder of the survey, participants were able to uncheck or leave survey items blank and still complete and submit the questionnaire.

The questionnaire was pilot tested on a minimum of 5 practising chiropractors from each country/region surveyed. Feedback was provided on survey content, wording and length that resulted in further refinements. The finalised online version took approximately 15 min to complete.

### Statistical analysis

Practitioners’ characteristics were described using frequencies with percentages or means with standard deviations (SD) as appropriate. Pearson’s chi-squared test was used to assess differences between regions in chiropractors’ public health response to and business/financial impacts from COVID-19.

Investigation of differences in COVID-19 patient management factors between musculoskeletal spine-care and subluxation-based paradigms was then performed using independent samples *t*-tests and chi-squared tests for continuous and categorical variables, respectively. Some categorical response items (e.g., level of face-to-face patient care during the peak of the COVID-19 outbreak, or personal income changes during the peak of the COVID-19 outbreak) were dichotomised as “increase” or “no change/decrease” for chi-squared analyses. Only items demonstrating significant between-group differences in the univariate analyses (*p* < 0.05) were entered into a multivariable logistic regression model. Backward elimination was used to identify items associated with chiropractors practising in a musculoskeletal spine-care paradigm after adjusting for country of practice and age. Statistical significance was set at *p* < 0.05. Odds ratios were reported with 95% confidence intervals (CI). Statistical analyses were conducted using *SPSS Statistics (*v27.0) *for Windows* (IBM Corp; Armonk, NY).

## Results

### Socio*-demographic findings*

In total, 2484 chiropractors participated, with 2061 (83%) fully completing the survey for analysis (Table 1). Our recruitment method did not allow the calculation of an accurate response rate. Practitioners’ average age was 47.5 (SD: 11.9) years with an average of 19.6 (SD: 11.4) years in practice, and 52% were male (n = 1061). The largest percentage of participating practitioners practised in Canada (35%), followed by the UK (23%), US (16%), Australia (13%), Denmark (7%) and Hong Kong (3%). Those practising outside these regions (1%) were identified as ‘Others’. Most participants practised in a town/small regional city (46%), followed by a major city (45%) and rural/remote location (8%). Most received their chiropractic education in the US (32%), followed by Canada (25%), UK (23%), Australia (14%) and Denmark (7%). Around two-thirds identified their practice paradigm as musculoskeletal spine-care and nearly one third as chiropractic subluxation-based care.

**Table 1:** Sociodemographic characteristics of participating chiropractors (n = 2061)

Characteristics	n (%)
*Gender (n* = *2061)*	
Male	1061 (51.5%)
Female	988 (47.9%)
Other/prefer not to say	12 (0.6%)
Age (Mean ± SD)	47.5 ± 11.9 years
Years in practice (Mean ± SD)	19.6 ± 11.4 years
*Primary practice region (n* = *2061)*	
Town or smaller regional city	954 (46.3%)
Major city (Urban/metropolitan)	935 (45.4%)
Rural/remote region	172 (8.3%)
*Country of practice*	
Canada	727 (35.3%)
United Kingdom	468 (22.7%)
United States	333 (16.2%)
Australia	279 (13.5%)
Denmark	153 (7.4%)
Hong Kong	69 (3.3%)
Others	28 (1.3%)
*Country of education*	
United States	654 (31.7%)
Canada	514 (24.9%)
United Kingdom	481 (23.3%)
Australia	294 (14.2%)
Denmark	84 (4.1%)
New Zealand	11 (0.5%)
Others	21 (1.0%)
*Highest professional qualifications*	
Doctor of Chiropractic	1496 (72.6%)
Bachelor/double Bachelor	325 (16.3%)
Master of Chiropractic	167 (8.1%)
Diploma/Advanced Diploma	63 (3.1%)
*Highest postgraduate qualifications*	
Master of Science	504 (24.5%)
Doctor of Philosophy	34 (1.6%)
More than one	1523 (73.9%)
*Practice setting (n* = *3483)*	
Other chiropractor(s) or osteopath(s)	1126 (32.3%)
Complementary medicine practitioners e.g., Massage therapist, Acupuncturist, Naturopath	1040 (29.8%)
Allied Health Practitioner e.g., Psychologist, Physical therapist, Exercise Physiologist, Podiatrist, Dietician	600 (17.2%)
Sole practitioner only	526 (15.1%)
GP/Family Physician or Medical Specialist	191 (5.5%)
*Practice paradigm (n* = *2061)*	
Spine/musculoskeletal conditions	1388 (67.3%)
Chiropractic subluxations	573 (27.8%)
Neither	100 (4.9%)

### Public health response to COVID-19 by chiropractors

Most participants reported implementing key COVID-19 infection control procedures within their practice setting, including the increased disinfection of treatment tables/equipment (95%) and frequent contact areas (94%), increased hand hygiene (94%), increased provision of hand sanitiser for patient use (93%) and increased social distancing of patients within the practice setting (89%) (Table 2).

**Table 2:** Practitioner and practice response to the COVID-19 pandemic (n = 2050)

	Overall n (% yes)	Australia n (% yes)	Canada n (% yes)	Denmark n (% yes)	Hong Kong n (% yes)	UK n (% yes)	USA n (% yes)	Others n (% yes)
*Changes within practice setting in response to the COVID-19 outbreak*								
Increased disinfecting of treatment table/equipment*	1942 (94.7%)	270 (96.8%)	704 (97.2%)	131 (85.6%)	61 (88.4%)	447 (96.1%)	301 (91.5%)	28 (90.3%)
Increased disinfecting of high contact areas*	1934 (94.3%)	264 (94.6%)	711 (98.2%)	146 (95.4%)	57 (82.6%)	436 (92.8%)	293 (89.1%)	27 (87.1%)
Increased disinfecting/cleaning of my hands*	1920 (93.7%)	265 (95.0%)	704 (97.2%)	134 (87.6%)	69 (100%)	435 (93.5%)	289 (87.8%)	24 (77.4%)
Providing patient hand sanitiser*	1909 (93.1%)	269 (96.4%)	724 (95.0%)	144 (94.1%)	62 (89.9%)	449 (96.6%)	266 (80.9%)	31 (100%)
Social distance patient seating*	1828 (89.2%)	246 (88.2%)	690 (95.3%)	142 (92.8%)	40 (58.0%)	405 (87.1%)	277 (84.2%)	28 (90.3%)
Change spacing patient bookings*	1629 (79.5%)	186 (66.7%)	663 (91.6%)	98 (64.1%)	23 (33.3%)	416 (89.5%)	223 (67.8%)	20 (64.5%)
Protective screen added to reception desk*	1202 (58.6%)	81 (29.0%)	549 (75.8%)	117 (76.5%)	36 (52.2%)	270 (58.1%)	134 (40.7%)	15 (48.4%)
Care restricted to urgent/emergency cases*	805 (39.3%)	67 (24.0%)	370 (46.0%)	75 (49.0%)	9 (13.0%)	202 (43.4%)	73 (22.2%)	9 (29.0%)
Patient care stopped*	764 (37.3%)	230 (17.6%)	351 (48.5%)	34 (22.2%)	1 (1.4%)	246 (52.9%)	71 (21.6%)	12 (38.7%)
None of the above	10 (0.5%)	0 (0.0%)	3 (0.4%)	0 (0.0%)	0 (0.0%)	2 (0.4%)	4 (1.2%)	1 (3.1%)
*Government/regulator advice on practitioner use of personal protective equipment (PPE) in response to COVID-19 (n = 2058)*								
Wearing cloth/standard surgical mask*	1755 (85.3%)	188 (67.4%)	670 (92.2%)	134 (87.6%)	65 (94.2%)	407 (87.2%)	289 (80.7%)	23 (74.2%)
Wearing face and/or eye shielding*	969 (47.1%)	23 (8.2%)	82 (11.3%)	9 (5.9%)	31 (44.9%)	82 (17.6%)	66 (19.9%)	8 (25.8%)
Wearing new disposable gloves during treatment*	723 (35.1%)	44 (15.8%)	90 (12.4%)	3 (2.0%)	50 (72.5%)	440 (94.2%)	88 (26.5%)	8 (25.8%)
Wearing protective garments/clothing*	673 (32.7%)	15 (5.4%)	180 (24.8%)	0 (0.0%)	10 (14.5%)	427 (91.4%)	39 (11.7%)	2 (6.5%)
Wearing N95 respirator/mask*	301 (14.6%)	23 (8.2%)	82 (11.3%)	9 (5.9%)	31 (44.9%)	82 (17.6%)	66 (19.9%)	8 (25.8%)
None of the above were advised*	86 (4.1%)	46 (16.5%)	15 (2.1%)	2 (2.9%)	2 (2.9%)	5 (1.1%)	12 (14.1%)	23 (74.2%)
Not sure what was advised*	61 (3.0%)	23 (13.6%)	8 (1.1%)	1 (0.7%)	1 (1.4%)	8 (1.7%)	19 (5.7%)	1 (3.2%)
Implementation of advised use of personal protective equipment (PPE) for chiropractors (n = 2025)*								
Yes, all that were advised (when available)	1611 (84.8%)	150 (58.8%)	603 (83.3%)	138 (91.4%)	64 (97.0%)	405 (86.9%)	233 (70.2%)	18 (58.1%)
Yes, some of what was advised	253 (12.9%)	40 (15.8%)	93 (12.8%)	10 (6.6%)	1 (1.5%)	48 (10.3%)	55 (16.6%)	6 (19.4%)
Never or rarely	161 (2.3%)	65 (25.5%)	28 (3.9%)	3 (2.0%)	1 (1.5%)	13 (2.8%)	44 (13.3%)	7 (22.6%)
*Government/regulator advice on patient use of personal protective equipment (PPE) in response to COVID-19 (n = 2057)*								
Wearing cloth/standard surgical mask*	1697 (82.5%)	160 (57.8%)	649 (89.3%)	135 (88.2%)	66 (97.1%)	380 (81.4%)	286 (85.9%)	21 (65.6%)
Wearing face and/or eye shielding*	189 (9.2%)	11 (4.0%)	35 (4.8%)	34 (22.2%)	42 (60.9%)	37 (7.9%)	28 (8.4%)	2 (6.3%)
Wearing an N95 respirator/mask*	119 (5.8%)	10 (3.6%)	23 (3.2%)	6 (3.9%)	31 (44.9%)	18 (3.9%)	29 (8.7%)	2 (6.3%)
Wearing new disposable gloves during treatment *	114 (5.5%)	2 (0.7%)	14 (1.9%)	0 (0.0%)	41 (59.4%)	42 (9.0%)	11 (3.3%)	4 (3.5%)
Wearing protective garments/clothing	61 (3.0%)	0.0 (0.0%)	17 (2.3%)	0 (0.0%)	3 (4.3%)	38 (8.1%)	3 (0.9%)	0 (0.0%)
None of the above were advised*	209 (10.2%)	76 (27.3%)	54 (7.4%)	7 (4.6%)	2 (2.9%)	48 (10.3%)	14 (4.2%)	8 (25.0%)
Not sure what was advised*	94 (4.6%)	32 (11.5%)	12 (1.7%)	1 (0.7%)	1 (1.4%)	23 (4.9%)	24 (7.2%)	1 (3.1%)
Implementation of advised use of personal protective equipment (PPE) for patients (n = 2016)*								
Yes, all that were advised (when available)	1524 (75.6%)	128 (52.2%)	597 (82.2%)	138 (91.4%)	64 (95.5%)	350 (75.6%)	230 (69.1%)	17 (54.8%)
Yes, some of what was advised	165 (8.2%)	24 (9.8%)	51(7.0%)	5 (3.3%)	2 (3.0%)	32 (19.4%)	47 (14.1%)	17 (54.8%)
Never or rarely	327 (16.2%)	93 (38.1%)	78 (10.7%)	8 (5.3%)	1 (0.3%)	82 (24.8%)	56 (16.8%)	10 (32.3%)
Approach to care recommended for patients presenting with COVID-19 or similar flu-like symptoms (n = 2056)*								
Avoid treatment until after testing -ve or after quarantine for at least 2 weeks if + ve	1698 (82.6%)	239 (86.3%)	612 (84.4%)	111 (72.5%)	60 (87.0%)	414 (88.7%)	238 (71.5%)	24 (75.0%)
Advise to avoid treatment until after their symptoms passed	237 (11.5%)	20 (7.2%)	87 (12.0%)	37 (24.2%)	3 (4.3%)	40 (8.6%)	45 (13.5%)	5 (15.6%)
Provide treatment using additional protective measures	90 (4.4%)	16 (5.8%)	19 (2.6%)	4 (2.6%)	4 (5.8%)	8 (1.7%)	39 (11.4%)	1 (3.1%)
Provide treatment without additional protective measures	19 (0.9%)	1 (0.4%)	4 (0.6%)	0 (0.0%)	2 (2.9%)	4 (0.9%)	7 (2.1%)	1 (3.1%)
Not sure what to do	12 (0.6%)	1 (0.4%)	3 (0.4%)	1 (0.7%)	0 (0.0%)	1 (0.2%)	5 (1.5%)	1 (3.1%)
Use of teleconferencing/telehealth before the COVID-19 outbreak began (n = 2051)*								
Yes	107 (5.2%)	12 (4.3%)	20 (2.8%)	9 (5.9%)	2 (1.9%)	35 (7.5%)	28 (8.4%)	1 (3.2%)
Use of teleconferencing/telehealth since the COVID-19 outbreak began (n = 1947)*								
Yes	515 (26.5%)	33 (12.4%)	176 (25.0%)	24 (16.7%)	1 (1.5%)	196 (45.4%)	76 (25.0%)	9 (29.0%)
Percentage of patient consultations provided through teleconferencing during the peak of the COVID-19 outbreak (n = 621)*								
More than 75%	157 (25.2%)	3 (6.7%)	39 (20.0%)	4 (12.1%)	1 (33.3%)	35 (7.5%)	28 (8.4%)	2 (20.0%)
Between 50–75%	19 (2.9%)	1 (5.6%)	5 (2.8%)	0 (0.0%)	0 (0.0%)	7 (3.0%)	5 (4.8%)	0 (0.0%)
Between 25–50%	29 (4.7%)	2 (4.4%)	7 (3.6%)	2 (6.1%)	1 (33.3%)	8 (3.5%)	9 (8.7%)	0 (0.0%)
Between 1–25%	263 (42.4%)	34 (75.6%)	105 (53.8%)	23 (69.7%)	1 (33.3%)	52 (22.5%)	45 (43.3%)	3 (30.0%)
Not in practise during peak	154 (24.8%)	5 (11.1%)	39 (20.0%)	4 (12.1%)	0 (0.0%)	84 (36.4%)	17 (16.3%)	5 (50.0%)
A lack of third-party financial funding as barrier to the use of teleconferencing during COVID-19 (n = 2042)*								
No	1154 (56.6%)	160 (57.8%)	468 (65.3%)	129 (84.9%)	52 (75.4%)	156 (33.5%)	174 (52.7%)	15 (46.9%)
Yes	328 (16.1%)	56 (20.2%)	113 (15.8%)	14 (9.2%)	4 (5.8%)	48 (10.3%)	90 (27.4%)	3 (9.4%)
Not relevant (all care is fully patient funded)	560 (20.6%)	61 (10.9%)	136 (19.0%)	9 (5.9%)	13 (18.8%)	261 (56.1%)	66 (20.0%)	14 (43.3%)
Independent guidelines needed for public health procedures for manual therapy practitioners (n = 2053)*								
No, same guidelines for all healthcare practitioners	936 (45.6%)	103 (37.1%)	332 (45.8%)	88 (58.3%)	51 (75.0%)	176 (37.7%)	175 (52.7%)	11 (34.4%)
Yes	848 (41.3%)	138 (49.6%)	276 (38.1%)	51 (33.8%)	15 (22.1%)	240 (51.4%)	113 (34.0%)	15 (46.9%)
Unsure	269 (13.1%)	37 (13.3%)	117 (16.1%)	12 (7.9%)	2 (2.9%)	51 (10.9%)	44 (13.3%)	6 (18.8%)

Some items with missing data ranging from 1 to 11 respondents, *P < 0.001

Responses varied by country on government/health regulator advice on practitioner use of PPE in response to COVID-19. The overall majority reported that wearing of a cloth or standard surgical mask had been advised (85%), while a smaller percentage (47%) reported that wearing face and/or eye shielding, wearing disposable gloves during treatment (35%), wearing protective clothing (33%) and wearing an N95 respirator/mask (15%) had been advised. Approximately 85% of chiropractors reported that they followed all that had been advised on practitioner use of PPE (when available), while 13% reported they followed some of what had been advised and 2% indicating that they never/rarely implemented recommendations for practitioners use of PPE.

Responses also varied by country on government/health regulator advice on chiropractic patient use of PPE in response to COVID-19. The overall majority reported that patient wearing of a cloth or standard surgical mask was advised (82%), while a smaller percentage (9%) reported wearing face and/or eye shielding, wearing an N95 respirator/mask (6%), wearing disposable gloves during treatment (5%) or wearing protective garments/clothing (3%) had been advised. Most chiropractors (76%) reported that they had followed all that had been advised on patient use of PPE (when available) while 8% reported they had followed some of what had been advised and 16% indicating they had never/rarely implemented patient use of PPE recommendations.

When participants were asked about the approach to care they would recommend for a patient presenting to their practice with COVID-19 or similar flu-like symptoms, 83% would recommend not providing treatment until the patient has tested negative and after quarantining for at least 2 weeks if testing positive. Approximately 11% recommended these patients not receive treatment until the symptoms had passed, while 4% would proceed with treatment using additional PPE.

Only 5% of participants reported using patient telehealth before the pandemic began, while 26% reported telehealth use after the outbreak of COVID-19. Telehealth most often contributed to ≤ 25% of overall patient consultations for those who utilised this modality during the peak of the pandemic. Only 16% reported that a lack of third-party reimbursement was a barrier to their use of telehealth. Participants were divided on whether the public health procedures for manual therapy participants warranted independent guidelines (41% in favour and 46% against) with 13% unsure.

Approximately 83% of participants reported providing COVID-19 public health information, such as hand hygiene, social distancing, oral hygiene and face mask wearing to patients during the pandemic (Table 3). This was most often provided during face-to-face consultations (78%), during patient phone calls (64%) and through brochures/posters placed inside the practice/entry areas (64%). Chiropractors’ most trusted resources for COVID-19 public health information were government reports/websites (42%), followed by chiropractic professional associations/organisations (29%).

**Table 3:** COVID-19 patient education provided by chiropractors during the COVID-19 pandemic (n = 2061)

	Overall n (% Yes)	Australia n (% Yes)	Canada n (% Yes)	Denmark n (% Yes)	Hong Kong n (% Yes)	UK n (% Yes)	USA n (% Yes)	Others n (% Yes)
Provided COVID-19 public health information to patients (such as hand washing, social distancing, oral hygiene, mask use or similar) (n = 2049)*								
Yes	1692 (82.6%)	229 (82.4%)	610 (84.8%)	142 (92.8%)	39 (56.5%)	383 (82.4%)	265 (79.6%)	24 (75.0%)
*Approach to providing public health information about COVID-19 to patients (n = 1692)*								
During face-to-face consultations	1319 (78.0%)	185 (80.8%)	471 (77.2%)	94 (66.2%)	29 (74.4%)	298 (77.8%)	228 (86.0%)	14 (58.3%)
On patient phone calls*	1088 (64.3%)	123 (53.7%)	399 (65.4%)	94 (66.2%)	27 (69.2%)	281 (73.4%)	153 (57.7%)	11 (45.8%)
Brochures/posters in practice areas*	1077 (63.7%)	150 (65.5%)	425 (69.7%)	110 (77.5%)	25 (64.1%)	226 (59.0%)	130 (49.1%)	11 (45.8%)
Through patient emails/mail-out*	872 (51.5%)	107 (46.7%)	356 (58.4%)	25 (17.6%)	16 (41.0%)	249 (65.0%)	108 (40.8%)	13 (45.8%)
On the practice website*	864 (51.1%)	98 (42.8%)	316 (51.8%)	105 (73.9%)	24 (61.5%)	224 (58.5%)	91 (34.3%)	6 (25.0%)
Social media posts*	744 (44.0%)	100 (43.7%)	253 (41.5%)	81 (57.0%)	15 (38.5%)	187 (48.8%)	98 (37.0%)	10 (41.7%)
Patient text-messages*	536 (31.7%)	93 (40.6%)	119 (19.5%)	69 (48.6%)	19 (48.7%)	158 (41.3%)	70 (26.4%)	8 (33.3%)
During patient teleconferencing/webinar*	252 (14.9%)	14 (6.1%)	59 (23.4%)	13 (5.2%)	18 (46.2%)	95 (24.8%)	50 (18.9%)	3 (12.5%)
*Most trusted resource for COVID-19 public health information (n = 2022)*								
Chiropractic associations/organisations	597 (29.5%)	88 (32.2%)	171 (23.8%)	26 (17.1%)	36 (52.2%)	215 (47.1%)	56 (17.4%)	5 (15.5%)
Chiropractic regulatory board	302 (14.9%)	21 (7.7%)	205 (23.8%)	1 (0.7%)	1 (1.4%)	54 (11.8%)	20 (6.2%)	0 (0.0%)
Government reports/website	846 (41.8%)	131 (48.0%)	287 (40.0%)	118 (77.6%)	23 (33.3%)	135 (29.6%)	136 (42.2%)	16 (50.0%)
World Health Organisation	75 (3.7%)	12 (4.4%)	14 (1.9%)	1 (0.7%)	7 (10.1%)	15 (3.3%)	22 (6.8%)	4 (12.5%)
Search/reviewing COVID-19 research myself	160 (7.9%)	16 (5.9%)	32 (4.5%)	3 (2.0%)	1 (1.4%)	30 (6.6%)	76 (23.6%)	2 (6.3%)
Preferred commentators (media/social media)	13 (0.6%)	1 (0.4%)	3 (0.4%)	0 (0.0%)	0 (0.0%)	2 (0.4%)	7 (2.2%)	0 (0.0%)
TV/internet/radio/Newspaper news reports	18 (0.9%)	4 (1.5%)	1 (0.1%)	3 (2.0%)	0 (0.0%)	2 (0.4%)	4 (1.2%)	4 (12.5%)
Family/friend/another chiropractor	11 (0.5%)	0 (0.0%)	5 (0.7%)	0 (0.0%)	1 (1.4%)	3 (0.7%)	1 (0.3%)	1 (3.1%)

Some items with missing data ranging from 1 to 11 respondents, *P < 0.001

### Impacts of COVID-19 on business and finances of chiropractors

The impacts of the COVID-19 pandemic on the business and finances of chiropractors are reported in Table 4. A complete suspension of face-to-face patient care during the peak of the pandemic was reported by almost half of all participants (49%), while 26% reported a greater than 50% decrease in face-to-face patient care compared to normal. Practice suspension was greatest in the UK (78%) and Canada (65%) and least in Hong Kong (1%) and Australia (11%). In terms of income, 43% reported that their personal income had completely stopped, while 27% reported that their income was reduced by more than 50% during the peak of the pandemic. During this time, 66% of chiropractors reported needing to seek financial assistance due to their loss of income. The majority also reported substantial negative impacts on the employment of other practice staff during the peak of the pandemic, with nearly half (49%) reporting temporary work leave, and nearly a third (30%) reported decreased staff work hours and/or income during the outbreak. When participants were asked how the COVID-19 outbreak might impact their patient care after the pandemic is over, most reported that the pandemic would likely have a lasting influence on the delivery of patient care, including continuing with more frequent disinfecting of practice equipment/areas (68%), increased use of hand sanitiser (57%), more frequent rescheduling of patients who present with flu-like symptoms (52%) and increased use of PPE (36%).

**Table 4:** Impacts of COVID-19 pandemic on the business and finances of chiropractors (n = 2058)

	Overall n (% Yes)	Australia n (% Yes)	Canada n (% Yes)	Denmark n (% Yes)	Hong Kong n (% Yes)	UK n (% Yes)	USA n (% Yes)	Others n (% Yes)
Change in level of face-to-face patient care during the peak of the COVID-19 outbreak*								
Practice suspended due to COVID-19 pandemic	1014 (49.3%)	32 (11.5%)	470 (64.6%)	34 (22.4%)	1 (1.4%)	363 (77.7%)	98 (29.4%)	16 (50.0%)
Decreased > 50%	528 (25.7%)	77 (27.7%)	190 (26.1%)	89 (58.6%)	3 (4.3%)	57 (12.2%)	108 (32.4%)	4 (12.5%)
Decreased 25–50%	203 (9.9%)	81 (29.1%)	27 (3.7%)	13 (8.6%)	9 (13.0%)	5 (1.1%)	62 (18.6%)	6 (18.8%)
Decreased 1–25%	103 (5.0%)	49 (17.6%)	9 (1.2%)	2 (1.2%)	5 (7.2%)	3 (0.6%)	33 (9.9%)	2 (6.3%)
Stayed about the same	49 (2.4%)	15 (5.4%)	5 (0.7%)	4 (2.6%)	6 (8.7%)	2 (0.4%)	15 (4.5%)	2 (6.3%)
Increased 1–25%	26 (1.3%)	8 (2.9%)	2 (0.3%)	0 (0.0%)	9 (13.0%)	1 (0.2%)	6 (1.8%)	0 (0.0%)
Increased 25–50%	19 (0.9%)	7 (2.5%)	2 (0.3%)	0 (0.0%)	7 (10.1%)	0 (0.0%)	3 (0.9%)	0 (0.0%)
Increased > 50%	39 (1.9%)	3 (1.1%)	1 (0.1%)	2 (1.3%)	27 (39.1%)	2 (0.4%)	4 (1.2%)	0 (0.0%)
Not practising during peak of COVID-19	77 (3.7%)	6 (2.2%)	6 (2.2%)	21 (2.9%)	8 (5.3%)	2 (2.9%)	34 (7.3%)	2 (6.3%)
Change in personal income as a chiropractor during the peak of the COVID-19 outbreak (n = 1977)*								
Completely stopped	843 (42.6%)	18 (6.7%)	420 (59.5%)	13 (9.0%)	1 (1.5%)	320 (73.9%)	54 (16.5%)	17 (56.7%)
Decreased > 50%	526 (26.6%)	70 (26.0%)	218 (30.9%)	68 (47.2%)	5 (7.5%)	69 (15.9%)	93 (28.4%)	3 (10.0%)
Decreased 25%-50%	250 (12.6%)	76 (28.3%)	38 (5.4%)	34 (23.6%)	7 (10.4%)	25 (5.8%)	64 (19.5%)	8 (20.0%)
Decreased 1%-25%	135 (6.8%)	55 (20.4%)	16 (2.3%)	14 (9.7%)	4 (6.0%)	6 (1.4%)	39 (11.9%)	1 (3.3%)
Stayed about the same	140 (7.1%)	30 (11.2%)	13 (1.8%)	15 (10.4%)	7 (10.4%)	8 (1.8%)	65 (19.8%)	2 (6.7%)
Increased 1%-25%	34 (1.7%)	13 (4.8%)	0 (0.0%)	0 (0.0%)	10 (14.9%)	0 (0.0%)	10 (3.0%)	1 (3.3%)
Increased 25%-50%	24 (1.2%)	6 (2.2%)	1 (0.1%)	0 (0.0%)	11 (16.4%)	4 (0.9%)	2 (0.6%)	0 (0.0%)
Increased > 50%	25 (1.3%)	1 (0.4%)	0 (0.0%)	0 (0.0%)	22 (32.6%)	1 (0.2%)	1 (0.3%)	0 (0.0%)
Financial assistance sought due to a loss of practice and/or personal income because of the COVID-19 outbreak (n = 2048)*								
Yes	1359 (66.4%)	116 (41.9%)	596 (82.4%)	83 (55.0%)	2 (2.9%)	357 (76.8%)	192 (58.0%)	13 (40.6%)*
*Impact of the COVID-19 outbreak on the employment of other practice staff (n = 2.058)*								
Temporary work leave, but still employed (with/without government support)*	1011 (49.1%)	70 (25.2%)	454 (62.4%)	105 (68.6%)	4 (5.8%)	269 (57.6%)	96 (28.9%)	13 (40.6%)
Decreased work hours and/or income*	627 (30.5%)	106 (38.1%)	239 (32.9%)	38 (24.8%)	9 (13.0%)	122 (26.1%)	104 (31.3%)	9 (28.1%)
Complete loss of employment*	324 (15.7%)	22 (7.9%)	180 (24.8%)	13 (8.5%)	1 (1.4%)	69 (14.8%)	36 (10.8%)	3 (9.4%)
No substantial changes*	298 (14.5%)	80 (28.8%)	33 (4.5%)	20 (13.1%)	49 (71.0%)	11 (2.4%)	103 (31.0%)	2 (6.3%)
Not relevant (as no other practice staff)*	352 (17.1%)	45 (16.2%)	92 (12.7%)	15 (9.8%)	7 (10.1%)	127 (27.2%)	54 (16.2%)	12 (37.5%)
*Impact of COVID-19 outbreak on care after the COVID-19 pandemic is over (n = 2060)*								
Increased disinfecting/cleaning of practice equipment/areas*	1413 (68.3%)	204 (73.1%)	533 (73.3%)	119 (77.8%)	43 (62.3%)	303 (64.9%)	186 (55.9%)	20 (62.5%)
Greater use of hand sanitiser*	1166 (56.6%)	169 (60.6%)	458 (63.0%)	106 (69.3%)	39 (56.5%)	247 (52.9%)	132 (39.6%)	15 (46.9%)
More rescheduling of patients with flu-like symptoms*	1066 (51.7%)	145 (52.0%)	447 (61.5%)	90 (58.8%)	14 (20.3%)	234 (50.1%)	122 (36.6%)	14 (43.8%)
Greater use of (PPE)*	740 (35.9%)	69 (24.7%)	319 (43.9%)	38 (24.8%)	32 (46.4%)	187 (40.0%)	88 (25.8%)	9 (28.1%)
More social distancing in reception/treatment areas*	661 (32.1%)	91 (32.6%)	268 (36.9%)	67 (43.8%)	20 (29.0%)	129 (27.6%)	77 (23.1%)	9 (28.1%)
No changes, back to normal*	292 (14.2%)	36 (12.9%)	71 (9.8%)	12 (7.8%)	22 (31.9%)	69 (14.8%)	75 (22.5%)	7 (21.9%)
More teleconferencing/telehealth patient care*	242 (11.7%)	21 (7.5%)	83 (11.4%)	10 (6.5%)	4 (5.8%)	72 (15.4%)	50 (15.0%)	2 (6.3%)
Unsure of changes after the pandemic is over*	249 (12.1%)	23 (8.2%)	85 (11.7%)	8 (5.2%)	3 (4.3%)	72 (15.4%)	54 (16.2%)	4 (12.5%)

*P < 0.001

### Factors associated with chiropractic practice paradigms

Table 5 shows the items that were associated with a musculoskeletal spine-care paradigm.

**Table 5:** Differences in characteristics between chiropractors practising under a musculoskeletal spine-care paradigm versus subluxation-based paradigm (n = 2061; 95.1% of all respondents)

Characteristics	Musculoskeletal spine-care n = 1388 n (%) /mean (SD)	Subluxation-based care n = 573 n (%) /mean (SD)
*Sociodemographic characteristics*		
Mean age (SD) years*	46.7 ± (11.8)	49.1 ± 12.1
Years of practice (years)*	18.8 ± 11.3	21.2 ± 11.6
Degree (e.g., Master’s or PhD)*	1388 (27)	573 (22)
*Practice setting characteristics*		
Sole Proprietor*	1386 (22)	573 (33)
Working with other chiropractors/osteopaths*	1386 (58)	573 (49)
Working with general practitioners/medical specialists^#^	1387 (11)	573 (7)
Working with allied health practitioners*	1386 (34)	573 (18)
Working with complementary healthcare practitioners*	1387 (55)	573 (41)
*Practice setting changes in response to the outbreak*		
Protective screen added at the reception desk*	1384 (62)	568 (51)
Social distancing with patient seating in the reception and/or treatment area*	1384 (90)	568 (87)
Changes to the spacing of patient bookings*	1384 (82)	568 (73)
Care restricted to emergency/urgent cases only*	1384 (43)	568 (31)
Patient care was stopped*	1384 (42)	568 (25)
*Knowledge of health regulator advice on chiropractors use of PPE*		
No necessity to wear protective clothing*	1386 (35)	573 (27)
*Implementation of advised PPE use for chiropractors*		
Implementing some/all advised PPE for chiropractors*	1363 (94)	563 (90)
*Knowledge of health regulator advice on patient use of PPE*		
Wearing a cloth/standard surgical mask*	1386 (84)	571 (79)
No necessity to wear an N95 respirator/mask*	1387 (95)	572 (91)
No necessity to wear face and/or eye shielding*	1387 (93)	572 (86)
No necessity to use disposable gloves* (n = vs)	1387 (96)	572 (90)
Deeming that patients do not need to use any kind of PPE*	1387 (9)	572 (14)
*Implementation of advised PPE use for patients*		
Implementing some/all advised PPE for patients*	1352 (87)	565 (77)
*Approach to patient presenting with COVID-19 or flu-like symptoms*		
No treatment until the patient had negative COVID-19 test results, completed the 14-day quarantine if positive, or had no more flu-like symptoms*	1385 (97)	573 (89)
*Use of teleconferencing/telehealth*		
Before COVID-19 outbreak*	1381 (5)	571 (3)
During COVID-19 outbreak*	1308 (32)	558 (14)
*Public health education*		
Providing public health education*	1195 (87)	421 (74)
Providing such education through their practice website*	1195 (54)	421 (44)
Providing such education through social media^#^	1195 (45)	421 (39)
*Most trusted sources for COVID-19 information*		
Information from authorities (e.g., national/regional government, chiropractic associations, World Health Organization, chiropractic registration/regulation boards)*	1368 (94)	556 (84)
*Support for public health independent guidelines for chiropractors*		
In favour of independent guidelines*	1385 (46)	570 (31)
*Face-to-face treatments during the peak of COVID-19 outbreak*		
Decreased as compared to before the outbreak*	1385 (97)	573 (86)
*Change in personal income during the peak of COVID-19 outbreak*		
The same or increased as compared to before the outbreak*	1332 (9)	558 (17)
*Seeking financial assistance during the peak of COVID-19 outbreak*		
Yes*	1381 (70)	568 (59)
*Anticipated changes in practice after the COVID-19 pandemic*		
Temporary leave from work but still employed*	1385 (53)	573 (41)
Decreased work hours/income*	1385 (32)	573 (26)
No substantial changes*	1385 (10)	573 (24)
*Changes in patient care after the COVID-19 pandemic*		
No change*	1387 (9)	573 (27)
Increased disinfecting/cleaning of practice equipment/area*	1387 (74)	573 (56)
Greater use of PPE*	1387 (42)	573 (25)
Greater use of hand sanitiser*	1387 (63)	573 (43)
More social distancing in reception and/or treatment areas*	1387 (36%	573 (25)
More rescheduling if patients have flu-like symptoms*	1387 (59)	573 (36)
More teleconferencing/telehealth patient care*	1387 (14)	573 (5)

All survey items demonstrating significant between-group differences: *P < 0.01; ^#^ P < 0.05

Factors associated with those practising under a musculoskeletal spine-care paradigm (as opposed to a subluxation-based paradigm) included practitioner sociodemographic background (age, sole practitioner, working with other healthcare professionals), public health knowledge regarding health regulator COVID-19 advice, public health response to COVID-19, public health views and business impacts (Table 6).

**Table 6:** Factors associated with chiropractors practising under a musculoskeletal spine-care paradigm identified from multivariable logistic regression

Factors	Unadjusted odds ratio (95% CI)	Adjusted odds ratio (95% CI)
*Demographics*		
Age (increase per additional year)	0.97 (0.96, 0.98)	0.99 (0.97, 1.00)
Sole practitioner	0.51 (0.40, 0.65)	0.64 (0.46, 0.90)
Working with medical doctors/specialists	2.24 (1.39, 3.63)	2.19 (1.05, 4.56)
Working with allied health practitioners	3.45 (2.53, 4.71)	1.94 (1.35, 2.78)
*Knowledge of government/health regulator advice and response to COVID-19*		
Believing government/health regulator advised the need for chiropractors to wear protective clothing	1.67 (1.28, 2.19)	1.56 (1.04, 2.34)
Implemented some/all government/health regulator advice on practitioner PPE use	1.94 (1.25, 2.98)	2.59 (1.32, 5.08)
Believing government/health regulator advised the need for patient use of standard surgical masks	1.69 (1.25, 2.30)	2.10 (1.04, 4.22)
Believing health regulator advised the need for patients to wear face and/or eye shielding	0.60 (0.41, 0.87)	0.47 (0.29, 0.76)
Implemented some/all government/health regulator advice on patient PPE use	2.36 (1.73, 3.21)	3.25 (1.57, 6.74)
No treatment until the patient had negative COVID-19 test results, completed the 14-day quarantine, or no more flu-like symptoms	4.73 (2.97, 7.54)	2.16 (1.18, 3.95)
Increased face-to-face care during the peak COVID-19	0.26 (0.16, 0.42)	0.36 (0.20, 0.65)
Initiate patient telehealth in response to COVID-19	3.26 (2.36, 4.50)	1.46 (1.02, 2.08)
*Need for independent public health guidelines for manual therapy providers*		
Yes	2.11 (1.64, 2.71)	1.33 (1.00, 1.76)
*Trusted resources when seeking public health information to guide clinical practice*		
Government reports/websites, World Health Organization, Chiropractic Registration Boards/Professional associations	3.05 (2.16, 4.31)	2.47 (1.49, 4.10)
*Employment status of other practice staff*		
No substantial change in their employment	0.41 (0.30, 0.55)	0.59 (0.39, 0.89)

All analyses are adjusted for country and age

## Discussion

A core function of healthcare providers is to limit the spread of COVID-19, both within their practice settings and through the promotion of evidence-based public health information. Variations in jurisdictional mandates for infection control will vary between regions, including in response to the ongoing regional changes in pandemic circumstances. This study primarily investigated the COVID-19 public health response of practicing chiropractors broadly (independent of specific regional standards at the time of data collection) and examined if paradigm predicts awareness and implementation of COVID-19 public health standards. Our study found that a substantial percentage of responding chiropractors, internationally, adopted a range of COVID-19 infection control measures in their practice settings and promoted COVID-19 public health information to their patients, to help reduce disease spread. These activities have the potential to assist wider government efforts to reduce the impacts of the pandemic on society. In addition, our findings show that the COVID-19 pandemic has had a substantial impact on the business and finances of chiropractors.

The infection control measures initiated by participants include increased disinfecting of practice areas and treatment equipment, increased personal hand sanitising, provision of patient hand sanitiser and the implementation of patient social distancing. The infection control measures used by respondents were mostly consistent with the information/resources advised by their professional regulatory bodies [15, 16] and this appears consistent with advice from government authorities and public health agencies (World Health Organisation and Centers for Disease Control and Prevention) [17, 18].

Interestingly, a much lesser percentage of Hong Kong chiropractors who participated reported implementing patient social distancing, changing the spacing of patient bookings and restricting patient care to emergency cases. While professional regulator links to government health directives in Hong Kong had included advice on social distancing [19] we speculate that these findings may reflect a comparatively reduced concern overall by Hong Kong practitioners at this early time point when the COVID-19 outbreak had been less severe to date and where no widespread government directed lockdowns nor stay-at-home orders had yet occurred.

The majority of participants reported implementing all the advised use of PPE provided by their respective government and/or health regulator. However, this majority was smaller for Australian (59%) and US (70%) chiropractors, which appears to suggest that chiropractors in these countries were more inconsistent in their approach to PPE recommendations despite recommendations made by health authorities [20–22] and major chiropractic professional associations providing links to COVID-19 health advice [23–25]. The low number of COVID-19 cases in Australia may be one reason for the relatively lower levels of PPE implementation by Australian chiropractors [26]. In addition, government advice on mask use at US state and federal levels has likely been more conflicting [27–29] and research has identified that this has influenced the level of mask use in different US regions [29]. Since, primary healthcare professionals in close physical contact with patients have an increased risk of COVID-19 cross infection without the appropriate use of PPE [3, 30], more research is needed to better understand the factors that influence the use of PPE by chiropractors, including the potential influence of contextual factors, such as the mainstream and social media [31, 32].

Our study found that the overall use of telehealth by chiropractors rose to one in four (26%) compared to one in twenty (5%) before the COVID-19 pandemic began. While this increase may be considered substantial for a profession that has traditionally relied on doing things 'by hand', by comparison, a near tenfold uptake of telehealth has been reported within other healthcare professions, such as medicine [33, 34] and physiotherapy [35]. Limited information does suggest that many chiropractors may have concerns that patient needs will not be met through telehealth [36]. However, evidence suggests the effectiveness of telehealth may be comparable to standard care for the management of spinal pain and other musculoskeletal conditions [37–40]. Importantly, many of the telehealth patient management strategies identified encompass approaches to patient care that appear to be commonplace within chiropractic settings, including patient education, advice on physical exercise, stress management, coping strategies and the use of pain medications [9, 41]. Our study findings also identified substantial regional variations in the use of telehealth. These variations may reflect differences in the occurrence of stay-at-home orders and closure of non-essential businesses between countries. For example, substantial periods of stay-at-home orders had occurred in the UK where the highest use of telehealth was reported (45%), while Australia (except for one state) [42] and Hong Kong [43] had experienced limited stay-at-home orders where the lowest use of telehealth was reported, at 12% and 1%, respectively. However, given and the knowledge that patients report telehealth appointments as helpful in addressing their concerns while providing a greater protection from COVID-19 transmission [44], more studies are needed to understand the limited comparative uptake of telehealth by chiropractors.

Chiropractors participating in the study were divided on whether the public health procedures for manual therapy practitioners warranted independent guidelines to those needed for other healthcare professionals, as found for dental settings [45]. Future research may therefore be needed to understand if chiropractors, and other manual therapy providers, present unique risks to the spread of infectious diseases. Such concerns have contributed, in-part, to the development of a recent guideline, led by chiropractic researchers, for the management of spinal disorders without face-to-face patient consultation in periods of mandated social distancing during a pandemic [46]. These guidelines advocate for patient consultation through telehealth for the management of uncomplicated spinal pain as well as providing practitioner guidance on the triage of patients with more serious underlying diseases.

More than 80% of surveyed chiropractors reported providing patients with public health information on COVID-19 infection control measures. This included during routine patient consultations, patient phone calls and through placing COVID-19 brochures and posters within their practice environment. The provision of reliable and accurate public health information by trusted healthcare professionals can improve patient protection from COVID-19 infection [47] and reduce confusion caused by COVID-19 misinformation [48] that undermines health authority advice, including scientifically proven treatments [49, 50]. Most chiropractors in our study identified recognised government agencies and professional bodies as their most trusted sources when seeking COVID-19 public health information. However, a smaller number of chiropractors have made claims on social media that chiropractic spinal manipulation reduces the adverse impact of COVID-19 [11, 12, 14], claims that appear to conflict with current clinical research evidence [51, 52]. In response, leaders of the chiropractic research community [53], chiropractic regulatory bodies [15, 16] and chiropractic professional associations [54–57] have made efforts to redress such claims within the profession.

Our study substantiated that the COVID-19 pandemic has had considerable negative impacts on the business and finances of chiropractors, which concurs with findings reported for other healthcare professions [5, 6]. Overall, nearly half of the participants reported a complete suspension of face-to-face patient care (49%) and around one in four (26%) reported a ≥ 50% decrease in face-to-face patient care during the peak of the pandemic. Additionally, around two-thirds reported needing to seek financial assistance because of their loss of income during the pandemic. However, practice suspension varied between countries, with the highest levels occurring in the UK (78%) and Canada (65%) and least in Hong Kong (1%) and Australia (11%). The relatively high proportion of chiropractors reporting a ≥ 50% decrease in face-to-face patient care could be attributed primarily to the responses from the UK and Canada. Findings also suggest seeking financial assistance was more frequent in those regions where a higher-level face-to-face patient care had ceased during the peak of the COVID-19 outbreak. Financial and employment uncertainty has been identified as an important contributor to healthcare workers’ stress and burnout during the COVID-19 pandemic [58–60]. In these circumstances, resolving the struggle of risking life or livelihood may not be an easy decision for many healthcare practitioners. However, the timely provision of government financial support, without discrimination or delays, is vital to healthcare practitioners during such rapidly changing employment conditions to help balance the potentially competing demands of business survival with adherence to public health policy objectives.

The practice paradigm study variable as operationalized in our survey did not allow the option for participants to self-select identifying with both paradigms in more equal measure, although chiropractors can practice under both paradigms as has been reported in other studies [61–63]. For public health responses collected in this study, participants who self-reported practising under a musculoskeletal spine-care paradigm differed from those reporting more closely practising under a chiropractic subluxation-based paradigm. This includes being more likely to adopt telehealth, demonstrate greater knowledge of regulator recommendations on the use of PPE, implement regulator advice on the use of PPE and not increase their face-to-face care during the peak of the COVID-19 outbreak. Since our study also found musculoskeletal spine-care chiropractors are more likely not to practice in a sole practitioner setting and are more likely to practice in multidisciplinary settings, it may be that multidisciplinary clinical settings foster greater knowledge translation of evidence-based public health initiatives. Such a finding has been identified in previous research [64], including for the control of COVID-19 infection [65].

Musculoskeletal spine-care chiropractors were also more likely to trust COVID-19 public health information provided by government, public health authorities and professional associations/boards—information sources with key responsibilities toward disseminating COVID-19 information based on current scientific consensus. This finding may also help to explain why musculoskeletal spine-care chiropractors are more likely to be aware of and implement certain COVID-19 infection control measures when compared to subluxation-based practitioners. It is vital that all chiropractors remain up to date with the advice provided by recognised health authorities to protect themselves and the public they serve during infectious disease outbreaks.

While our study identified that subluxation-based chiropractors constitute a smaller percentage of practitioners, more research is needed to understand how self-report practice paradigms may influence the public health knowledge and behaviours of chiropractors. Findings from this research may assist any knowledge translation strategies or infection control training for chiropractors if necessary to reduce the risk of communicable disease transmission, such as COVID-19, within chiropractic settings [66].

### Strengths and limitations

The study is the first chiropractic COVID-19 study to be conducted across several international regions. However, this study has several limitations. Self-reported data collection is subject to recall bias which may be further influenced by the timing of the survey relative to the previous peaks COVID-19 spread in the different countries. Those who self-selected to participate in the study may be different to non-participants and there is a risk of social desirability bias in the answers provided by those who did participate. The generalizability of the study is unknown because it was not possible to report the survey response rate accurately with an unknown number of chiropractors who viewed the survey preventing a precise assessment and acknowledge that the raw data should not be assumed to be representative of the greater population. This includes how often the survey was shared via social media connections and through personal emails and because the survey was not shared as a single email message, but most often embedded within another email within other online pages of information by chiropractic associations and professional online magazine. The reasons for why some chiropractors choose to participate and others did not, and the reasons for why some participants only partially completed the survey is unknown [67, 68]. There are many reasons why an individual may choose to not participate in this type of research. While difficult to speculate, we imagine some of these reasons may include: time constraints; forgetting to complete the survey or distraction, inadequate internet access, lack of interest in the topic; negative views toward COVID-19 or survey questions; concern about the security or confidentiality of the information that might be revealed during the survey; distrust of the motives of the researchers undertaking the survey; disinterest due to lack of direct personal benefit from participation (e.g., perceived value of the time necessary to complete the survey does not the outweigh the perceived potential benefit).

Further, representativeness of the sample is unknown because there was no reliable demographic information for chiropractors in most countries surveyed, hence non-response bias was unable to be measured. Response bias may exist because the survey dissemination was limited to those with email and internet access and to members of the professional associations and subscribers of the professional magazine utilised for the survey distribution and the unequal representation of participants across geographical regions and regulatory frameworks. Aside from face validity, items in this survey have not been assessed for their property measurements, including the question used for the identification of practice paradigm, which was limited to only three options i.e., those more closely musculoskeletal or subluxation-based, or neither. This leaves the possibility of additional paradigm subgroups to exist that are not accounted for in this study, such as chiropractors who practice more equally under both paradigms. This lack of choice may have impacted our response rate and representativeness of our survey since our respondents may not sufficiently represent the true self-identity professed by some practitioners within the general chiropractic population, as reported in other studies [61–63]. Therefore, the differences found between paradigms must be interpreted with considerable caution. While it was beyond the scope of this study to report on the exact mandates within each jurisdiction, this study has examined practitioner’s perceptions of what mandates were important to them and how they responded.

## Conclusion

Due to likely selection bias of our sample, we do not have confidence that these results are representative of the wider chiropractic population. Chiropractors in Australia, Canada, Denmark, Hong Kong, the UK and the US who participated in the study initiated a range of COVID-19 infectious control measures within their practice settings and have been providing patients with COVID-19 public health information. However, the implementation of telehealth and use of PPE was less uniform, warranting further research. Similar to other healthcare providers, COVID-19 has had a substantial negative business and financial impacts upon chiropractors. Finally, chiropractors who practice under a musculoskeletal spine-care paradigm were more likely to have knowledge of and implement several COVID-19 public health infection control measures important to reducing disease transmission compared to chiropractors practicing under a subluxation-based paradigm. It is vital that all healthcare providers implement and promote evidence-based public health behaviours to reduce the spread of COVID-19.

## Supplementary Information

**Additional file 1**. Practitioner survey questionnaire.

## Data Availability

The datasets used and/or analysed during the current study are available from the corresponding author on reasonable request.
