# Supplementary material for: The impact and public health response of chiropractors to the COVID-19 pandemic: a survey across four continents
Source: Chiropr Man Therap. 2022 May 9;30:24. doi: 10.1186/s12998-022-00432-6 (PMC9081962; doi:10.1186/s12998-022-00432-6)
Supplement: Supplementary file 1 — Additional file 1. Practitioner survey questionnaire. [file 12998_2022_432_MOESM1_ESM.doc]

**Chiropractic COVID-19 Global Impact Survey**

**Background**

1. **Your demographic and practice background**
2. Where do you primarily practice as a chiropractor? (Select one)
   1. Australia
   2. Canada
   3. Denmark
   4. Hong Kong SAR, China
   5. United Kingdom
   6. United States of America
   7. Other (If selected, please comment):
3. What is your age in years? (Enter whole number only)
4. How many years have you been in practice as a chiropractor? (Enter whole number)
5. What is your gender?
   1. Male
   2. Female
   3. Other
   4. Prefer not to say
6. Which of the following best describes the ***practice location*** where you primarily work as a chiropractor? (Select one)
   1. Rural/remote region
   2. Town or smaller regional city
   3. Major city (Urban/metropolitan/suburban)
7. Please provide the name of the institution where you received your chiropractic education (Please write in full for clarity)

[text box]

1. Which of the following educational qualifications do you hold? (Select all that apply).
   1. Diploma/Advanced Diploma
   2. Bachelor/double Bachelor
   3. The degree - Doctor of Chiropractic
   4. Master’s degree
   5. PhD
   6. Other (please describe)
2. Indicate any other healthcare professionals who work within the practice setting where you primarily work as a chiropractor (Select all that apply).
   1. Sole practitioner only
   2. Other chiropractor(s) or osteopath(s)
   3. General Practitioner (GP)/Family Physician or Medical Specialist
   4. Allied Health Practitioner e.g., Psychologist, Physiotherapist/Physical therapist, Exercise Physiologist (EP), Podiatrist, Dietician
   5. Complementary healthcare practitioners e.g., Massage therapist, Acupuncturist, Naturopath, Counsellor
   6. Other (please describe)
3. Which of the following statements would ***more closely*** describe the care you provide as a chiropractor (select one)?

a. Care based upon the detection and correction of chiropractic subluxations

b. Care based on the management of non-surgical spine and/or musculoskeletal conditions

d. Neither of the above

1. **Impacts of COVID-19 on practice-life**
2. Which of the following changes have occurred within your practice setting in response to the COVID-19 outbreak? (Select all that apply)
   1. Increased disinfecting/cleaning of my hands
   2. Provision of hand sanitizer for patients
   3. Increased disinfecting cleaning of treatment table/equipment
   4. Increased disinfecting/cleaning of frequent contact/reception areas
   5. Protective screen added at the reception desk
   6. Social distancing with patient seating in the reception and/or treatment area
   7. Changes to the spacing of patient bookings
   8. Care restricted to emergency/urgent cases only
   9. Patient care was stopped
   10. None of the above
3. What personal protective equipment (PPE) have ***chiropractors*** been ***advised to use*** by the government and/or regulator when providing patient care in response to the COVID-19 outbreak? (Select all that apply if the use has been advised, ***either*** generally or in particular clinical circumstances)
   1. Wearing a cloth/standard surgical mask
   2. Wearing an N95 respirator/mask
   3. Wearing face and/or eye shielding
   4. Wearing new disposable gloves during treatment for each patient
   5. Wearing protective garments/clothing
   6. None of the above were advised (jump to #13)
   7. Not sure what was advised (jump to #13)
4. Did the practice where you primarily work as a chiropractor seek to implement all the ***advised*** personal protective equipment (PPE) for chiropractors?
   1. Yes, all that were advised (depending on availability)
   2. Yes, some of what was advised
   3. No or rarely
5. What personal protective equipment (PPE) have ***chiropractic patients*** been ***advised to use*** by the government and/or regulator when ***receiving*** care in response to the COVID-19 outbreak? (Select all that apply if the use has been advised, either generally or in particular clinical circumstances)
   1. Wearing a cloth/standard surgical mask
   2. Wearing an N95 respirator/mask
   3. Wearing face and/or eye shielding
   4. Wearing new disposable gloves during treatment
   5. Wearing protective garments/clothing
   6. None of the above were advised (jump to #15)
   7. Not sure what was advised (jump to #15)
6. Did the practice where you primarily work as a chiropractor seek to implement all the ***advised*** personal protective equipment (PPE) for patients?
   1. Yes, all that were advised (depending on availability)
   2. Yes, some of what was advised
   3. No or rarely
7. What approach to care would you recommend for a patient who presents to your practice with COVID-19 or similar flu-like symptoms? (Select one)
   1. I would proceed with providing treatment without using any additional protective measures
   2. I would advise them not to have treatment until after they have been tested and after they had quarantined for at least 2 weeks if found to be positive.
   3. I would advise them not to have treatment until after their symptoms have passed
   4. I would proceed with providing treatment using additional protective measures
   5. I’m unsure what I would do
8. Were you already providing patient consultations through teleconferencing/telehealth ***before*** the COVID-19 outbreak began?
   1. Yes (jump to #18)
   2. No
9. Have you initiated patient consultations using teleconferencing/telehealth ***as a result*** of the COVID-19 outbreak?
   1. Yes
   2. No (jump to #19)
10. What percentage of your patient consultations were provided through teleconferencing/telehealth ***during the worst weeks*** of the COVID-19 outbreak in your region? (Select one)
    1. More than 75%
    2. Between 50%-75%
    3. Between 25%-50%
    4. Between 1%-25%
    5. I was not in practice during the worst weeks of COVID-19
11. Has a lack of financial funding of chiropractic consultations through teleconferencing, either from government or health insurance co-payments, ***been a barrier/restricted*** how often you have used teleconferencing during the COVID-19 outbreak?
    1. Yes
    2. No
    3. Not relevant (all chiropractic care in my region is fully patient funded only)
12. Have you or your practice been providing public health information about COVID-19 to your patients (such as hand washing, social distancing, oral hygiene, wearing a mask or similar)?
    1. Yes
    2. No (Jump to #22)
13. How have you or your practice provided public health information about COVID-19 to patients? (Select all that apply)
    1. During face-to-face consultations
    2. On the practice website
    3. During patient teleconferencing and/or webinars
    4. On patient phone calls
    5. Through patient emails/mail-outs
    6. Through patient text-messages
    7. Brochures and/or posters inside the practice and/or practice entry areas
    8. Through social media posts e.g., YouTube, Facebook, Instagram, twitter etc
14. What has been your most trusted resource when seeking out public health information to guide ***clinical practice procedures*** during the COVID-19 outbreak? (Rank in order from most to least trusted)
    1. National and/or regional government reports/websites
    2. Information provided via chiropractic professional associations/organisations
    3. World Health Organisation information
    4. Information from family members and/or friends and/or other chiropractors
    5. Preferred independent commentators (radio and/or television and/or social media)
    6. News reports on television/internet/radio/newspapers
    7. Information provided by chiropractic registration/regulatory boards
    8. Searching and reviewing relevant COVID-19 research myself
15. Given the close physical contact between patients and manual therapy practitioners (such as chiropractors), do you think there is a need for ***independent guidelines*** to help guide the public health procedures needed for this practitioner group in the future?
    1. Yes
    2. No, guidelines should be the same for all healthcare practitioners
    3. Unsure
16. **Impacts of COVID-19 on finances**
17. How much did your level of face-to-face patient care change ***during the worst weeks*** of the COVID-19 outbreak in your region? (Select one)
    1. Practicing was suspended because of COVID-19
    2. Decreased greatly (>50%)
    3. Decreased somewhat (25%-50%)
    4. Decreased slightly (1%-25%)
    5. Stayed about the same
    6. Increased slightly (1%-25%)
    7. Increased somewhat (25%-50%)
    8. Increased greatly (>50%)
    9. I was not in practice during the worst weeks of COVID-19 for other reasons
18. How has your personal income from practicing as a chiropractor changed ***during the worst weeks*** of the COVID-19 outbreak in your region? (Select one)
    1. My personal income completely stopped because of COVID-19
    2. Decreased greatly (>50%)
    3. Decreased somewhat (25%-50%)
    4. Decreased slightly (1%-25%)
    5. Stayed about the same
    6. Increased slightly (1%-25%)
    7. Increased somewhat (25%-50%)
    8. Increased greatly (>50%)
19. Have you needed to seek financial assistance (of any kind) due to a loss of practice and/or personal income because of the COVID-19 outbreak?
    1. Yes
    2. No
20. What has been the impact of the COVID-19 outbreak on the employment of ***other practice staff*** where you primarily practice as a chiropractor? (Select all that apply)
    1. Temporary leave from work, but still employed (with or without government support)
    2. Decreased work hours and/or income
    3. Complete loss of employment
    4. No substantial changes (to date)
    5. Not relevant (as we do not have any other practice staff)
21. How do you think the COVID-19 outbreak will impact/change how you care for patients ***after*** the pandemic is over? (Select all that apply)
    1. No changes, back to normal
    2. Increased disinfecting/cleaning of practice equipment/areas
    3. Greater use of personal protection equipment (PPE)
    4. Greater use of hand sanitizer
    5. More social distancing in reception and/or treatment areas
    6. More rescheduling of patients who have flu-like symptoms
    7. More teleconferencing/telehealth patient care
    8. I am unsure of what changes I will make after the pandemic is over
22. **Impacts of COVID-19 on your personal life (final few questions)**
23. Which of the following statements apply to your home environment ***during*** the COVID-19 outbreak? (Select all that apply)
    1. Living alone
    2. Living with a partner and/or other adults ***under*** the age of 65 years
    3. Living with partner and/or other adults ***over*** the age of 65 years
    4. Living with children under the age of 18 years.
    5. Living with a higher personal risk of COVID-19 because of an existing chronic health condition
    6. Living with someone else with a higher risk of COVID-19 because of an existing chronic health condition
    7. Living with someone with a disability
    8. None of the above
24. What have been your greatest concerns related to the COVID-19 outbreak? (Only select your ***3 greatest*** concerns from the list)
    1. Personal and family health concerns
    2. Community health concerns
    3. Concerns about the ability of my local hospital/medical clinic to manage COVID-19 patients
    4. Concerns about when there will be a return to regular daily practice without COVID-19 restrictions
    5. Concerns about having a coordinated response by authorities to COVID-19
    6. Concerns about when there will be a return to non-essential activities without COVID-19 related restrictions
    7. Concerns about loss of practice/personal income
    8. Concerns about loss of employment
    9. I have not had any concerns related to the COVID-19 outbreak
25. Have you been tested for COVID-19?
    1. Yes
    2. No (jump to #37)
    3. Prefer not to say (jump to #37)
26. What was the main reason for why you had a COVID-19 test? (Select one)
    1. I showed signs/symptoms similar to COVID-19
    2. I was in close contact with a chiropractic patient who had symptoms and/or tested positive for COVID-19
    3. I was in close contact with someone else (non-patient) who tested positive for COVID-19
    4. I was following a government and/or medical recommendation
    5. Because of my own personal concerns/reasons about COVID-19
    6. Other reason (not listed)
27. Did you stop working in clinical practice as a chiropractor between testing and finding out your test result?
    1. Yes
    2. No
    3. Prefer not to say
28. Have you tested positive for COVID-19?
    1. Yes
    2. No (jump to #37)
    3. Prefer not to say (jump to #37)
29. Did testing positive stop you from working in clinical practice as a chiropractor?
    1. Yes
    2. No
    3. Prefer not to say
30. Since you tested positive, were potentially exposed chiropractic patients then contacted and informed about this information?
    1. Yes
    2. No
    3. Unsure
    4. Prefer not to say
31. Finally, please feel free to write any comment you may have related to the COVID-19 outbreak or about this survey, otherwise please now press submit [free text field].

**Thank you for taking the time to complete this questionnaire.**
